# Retraction: Stopping the Revolving Door: Reducing 30-Day Psychiatric Readmissions With Post-discharge Telephone Calls

**DOI:** 10.7759/cureus.r40

**Published:** 2022-01-14

**Authors:** Antonia Phillip, Garrett Rossi, Roshi DeSilva

**Affiliations:** 1 Psychiatry, AtlantiCare Regional Medical Center, Galloway Township, USA

This article has been retracted at the request of the authors due to miscommunication among them which resulted in a lack of consent from the lead author to submit and publish this article. This author was also not provided an opportunity to review, contribute to, and approve the revisions prior to publication. As a result, Cureus has chosen to acquiesce to this request and retract the article.

